# Effects of suberoylanilide hydroxamic acid (SAHA) combined with paclitaxel (PTX) on paclitaxel-resistant ovarian cancer cells and insights into the underlying mechanisms

**DOI:** 10.1186/s12935-014-0112-x

**Published:** 2014-11-26

**Authors:** Zhaohui Liu, Ying Tong, Yuanlin Liu, Huaping Liu, Chundong Li, Yue Zhao, Yi Zhang

**Affiliations:** 1 Department of Obstetrics and Gynecology, Air Force General Hospital, Beijing, 100142 China; 2 Department of Cell Biology, Institute of Basic Medical Sciences, Academy of Military Medical Sciences, Beijing, 100850 China

**Keywords:** Suberoylanilide hydroxamic acid, Histone deacetylase inhibitors, Paclitaxel, Synergistic, Antitumor, Apoptosis, Cell-cycle arrest, Resistance, Chemotherapy

## Abstract

**Background:**

Suberoylanilide hydroxamic acid (SAHA) is a member of the hydroxamic acid class of the newly developed histone deacetylase inhibitors. Recently, Suberoylanilide hydroxamic acid has attracted increasing attention because of its antitumor activity and synergistic effects in combination with a variety of traditional chemotherapeutic drugs. Paclitaxel (PTX), is a natural anticancer drugs; however, resistance to paclitaxel has become a major challenge to the efficacy of this agent. The purpose of this study was to investigate the effects of the combined application of these two drugs on the paclitaxel-resistant ovarian cancer OC3/P cell line.

**Methods:**

In the present study, the effects of Suberoylanilide hydroxamic acid or/and paclitaxel on OC3/P cells cultured in vitro were analyzed in terms of cell viability, migration, cell-cycle progression and apoptosis by CCK-8, wound healing and flow cytometry assays. Changes in cell ultrastructure were observed by transmission electron microscopy. The expression of genes and proteins related to proliferation, apoptosis and drug resistance were analyzed by quantitative real-time polymerase chain reaction and Western blot analyses.

**Results:**

There was no cross-resistance of the paclitaxel-resistant ovarian cancer OC3/P cells to Suberoylanilide hydroxamic acid. Suberoylanilide hydroxamic acid combined with paclitaxel significantly inhibited cell growth and reduced the migration of OC3/P cells compared with the effects of Suberoylanilide hydroxamic acid or paclitaxel alone. Q-PCR showed the combination of Suberoylanilide hydroxamic acid and paclitaxel reduced intracellular *bcl-2* and *c-myc* gene expression and increased *bax* gene expression more distinctly than the application of SAHA or paclitaxel alone. Moreover, the level of *mdr1* gene expression in cells treated with Suberoylanilide hydroxamic acid was lower than that of the control group (*P* <0.05). Western blot analysis showed that Suberoylanilide hydroxamic acid alone or in combination with paclitaxel enhanced caspase-3 protein expression and degraded ID1 protein expression in OC3/P cells.

**Conclusion:**

Suberoylanilide hydroxamic acid inhibited the growth of paclitaxel-resistant ovarian cancer OC3/P cells and reduced migration by the induction of cell-cycle arrest, apoptosis and autophagy. These observations indicate the possible synergistic antitumor effects of sequential Suberoylanilide hydroxamic acid and paclitaxel treatment.

## Background

Epithelial ovarian cancer is the most lethal gynecologic malignancy [1]. Because of onset concealment, most patients are diagnosed in the middle or late stages of the disease, which makes combination chemotherapy the major strategy for ovarian cancer treatment. Although traditional combination chemotherapy, such as paclitaxel (also known as Taxol) combined with cisplatin, has improved the prognosis of the initial treatment of ovarian cancer, the 5-year survival rate of advanced-stage ovarian cancer is still between 15% and 20%, due to extensive primary and secondary drug resistance [2-6]. Therefore, the identification of new sensitive drugs and chemotherapy optimization programs for ovarian cancer in cases of chemotherapy resistance is imperative.

Although the molecular mechanisms underlying the occurrence and development of ovarian cancer are poorly understood, it has been confirmed that the occurrence of this type of cancer is closely associated with gene mutation, oncogene amplification and tumor suppressor gene deficiency [7]. With the development of epigenetics research, DNA methylation and histone acetylation have been shown to play important roles in tumor development [8], and they have become potential targets for chemotherapeutic intervention. HDAC inhibitors (HDACIs), which have attracted increasing attention in cancer drug development, exert anticancer activities by inhibiting cell proliferation and inducing cell-cycle arrest and apoptosis [9-11]. Suberoylanilide hydroxamic acid (SAHA) is a HDACI that shows strong anti-proliferative effects on various cancer cell lines and is currently in clinical trials for the treatment of certain solid and hematological tumors [12-15]. Moreover, SAHA is currently FDA-approved only for the treatment of progressive or recurrent cutaneous T-cell lymphoma (CTCL) for which the systematic treatments have failed [16]. In addition, SAHA shows sensitizing and synergistic effects with a variety of traditional chemotherapic drugs [17-19]. In particular, SAHA has been shown to inhibit the survival of sensitive ovarian cancer, and to have synergistic effects in combination with decitabine and paclitaxel [18-23]. There are currently no reports of systematic studies on the mechanism of the effects mediated by SAHA, alone and in combination with PTX, on paclitaxel-resistant ovarian cancer cell lines.

The specific goal of this study was to evaluate the anticancer effects of PTX or SAHA alone or in combination on OC3/P cells by investigating on cell viability, cell migration and invasion, cell ultrastructure, cell-cycle and apoptosis. Changes in the expression of genes and proteins related to apoptosis and drug resistance were also evaluated. Our study showed that SAHA inhibited the growth of OC3/P cells and reduced migration by the induction of cell-cycle arrest, apoptosis and autophagy. These observations indicate the possible synergistic antitumor effects of sequential SAHA and PTX treatment; however, further investigation of the functional mechanism should be carried out.

## Results

### Differences in biological properties between OC3 and OC3/P

**Figure 1:**
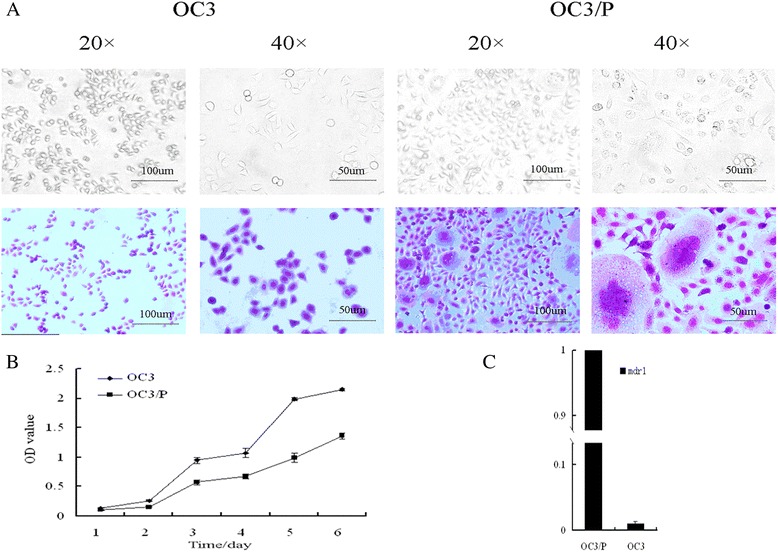
**Biological properties of the OC3 and OC3/P cell lines. A**: morphology of two cell lines viewed by inverted light microscopy (original magnification, ×20 and × 40). **B**: OC3 and OC3/P cell growth curves. Cell viability was determined with the CCK-8 assay every 24 h, for 6 days. **C**: Basal levels of mdr1 mRNA analyzed by Q-PCR. Relative expression was calculated following normalization to GAPDH levels.

As shown in Figure 1A, the aberrant nuclei were more prevalent among OC3/P cells than OC3 cells. The appearance of the OC3/P cells was mainly fusiform compared to the rounded shape of the OC3 cells. Growth curves for the two cell lines are shown in Figure 1B. The growth doubling-time of the OC3 cell line was approximately 27 h compared with 38 h for the OC3/P cell line. Moreover, *mdr1* expression in OC3/P was approximately 100 times greater than that in OC3 (Figure 1C). The IC_50_ values of the OC3 and OC3/P cell lines and the RI of OC3/P are shown in Table 1.

**Table 1:** **The RI and IC50s of two kinds of cells**

**Cell lines**	**IC50(μM)**		**RI**
	**24 h**	**48 h**	
OC3	2.74 ± 0.29	0.29 ± 0.24	1
OC3/P	28.43 ± 3.70	3.02 ± 1.67	10.15 ± 0.33

(n = 3) ($$ \overline{\mathrm{x}} $$ ±S).

IC50: half maximal inhibitory concentration, RI: resistance index.

### Viability of OC3 and OC3/P treated with SAHA or PTX

**Figure 2:**
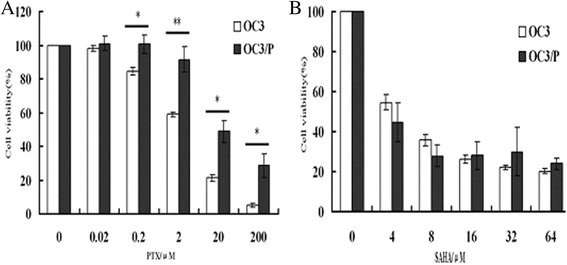
**Viability of OC3 and OC3/P cell lines treated with PTX or SAHA. A**: Viability of OC3 and OC3/P treated with various concentrations of PTX for 24 h. **P <0.01, *P <0.05. **B**: Viability of OC3 and OC3/P treated with various concentrations of SAHA for 48 h. No significant differences were observed between OC3 and OC3/P cell viability at any of the dose (*P* >0.05), implying that OC3/P is not cross-resistant to SAHA. Data represents the mean of three independent experiments. Error bars indicate one standard deviation from the mean.

The viabilities of the paclitaxel-sensitive and paclitaxel-resistant ovarian cancer cells (OC3 and OC3/P, respectively) treated with SAHA or PTX were compared. Both drugs exerted a concentration-dependent cytotoxic effect on both cell lines (Figure 2). The PTX-mediated growth inhibition of the sensitive cell line (OC3) was significantly greater than that of the resistant cell line (OC3/P) over the concentration range from 0.2 μM to 200 μM (Figure 2A; *P* <0.05). There was no significant difference in the viabilities of the two cell lines during a 48-h culture in the presence of 4, 16, 64 μM SAHA (Figure 2B; *P* >0.05).

### Effects of SAHA combined with PTX on cell growth and migration capability

**Figure 3:**
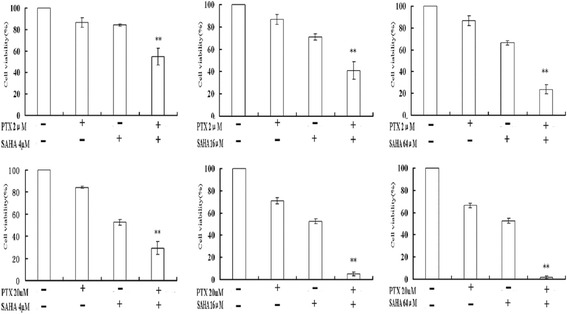
**Viability of OC3/P cells treated with SAHA or/and PTX.** Values represent the mean of three separate experiments. Error bars indicate one standard deviation from the mean. The experimental method was denoted in Cell viability of methods. **indicates a significant difference (the combination of SAHA and PTX compared with treatment with each individual agent) where *P* <0.01.

In every set of experiments, combined treatment with SAHA and PTX resulted in a significantly more pronounced reduction in cell viability compared with SAHA or PTX treatment alone (Figure 3).The viability of OC3/P treated with 2 μM PTX for 24 h was (91.70 ± 6.17)%, which was not significantly different from that of the control group (*P* >0.05). The viability of OC3/P treated with SAHA at 4, 16 and 64 μM for 24 h was (84.31 ± 0.81)%, (71.18 ± 2.83)% and (66.42 ± 1.89)%, respectively. However, the viability of cells pretreated with SAHA at these concentrations for 24 h followed by culture with 2 μM PTX medium for a further 24 h was (54.75 ± 7.54)%, (40.86 ± 7.77)% and (23.73 ± 4.43)%, respectively. These results also indicated the potential of SAHA for the reversal of drug resistance.

**Figure 4:**
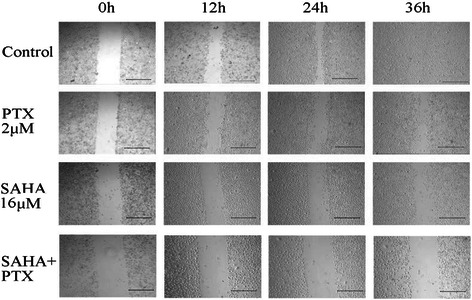
**Wound healing assays to assess the migration and invasion capacity of OC3/P cells treated with SAHA and/or PTX.** OC3/P cells in the exponential growth phase were trypsinized and plated into 6-well plates (4 × 10^4^cells/well). After incubation at 37°C under 5% CO_2_ for 24 h, cells were treated with PTX or SAHA alone or in combination (The pretreatment time and the details of co treatment was same with way in Cell viability of methods). A wound was made by scraping the cell monolayers with a 200 μl micropipette tip. Photographs were taken immediately and after 12 h, 24 h and 36 h using an IX70 microscope (OLYMPUS, Japan). (Scale bar, 500 μm).

The effects of SAHA or/and PTX on cell migration and invasion were determined with scratch wound healing assays. After combined treatment with SAHA and PTX, no migration of OC3/P cells occurred within 36 h, while varying degrees of cell migration occurred in the groups treated with SAHA or PTX alone (Figure 4). These results suggested that SAHA combined with PTX reduced cell migration and invasion capacity than SAHA or PTX treatment alone.

### Effects of SAHA combined with PTX on autophagy and cell-cycle

**Figure 5:**
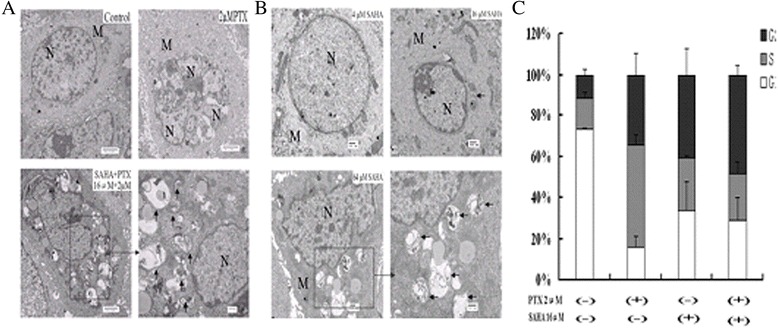
**Autophagy and cell-cycle regulation of OC3/P cells treated with SAHA or/and PTX. A**: Cells were pretreated with 16 μM SAHA for 24 h and then cultured with 2 μM PTX for a further 24 h, or were treated with serum-free 1640 for 24 h followed by PTX 1640 dilution for 24 h. Cell morphology and structure were viewed by transmission electron microscopy. **B**: Cells were treated with 4, 16 and 64 μM SAHA for 24 h followed by serum-free 1640 for 24 h. Cell morphology and structure were viewed by transmission electron microscopy. The arrows indicate autophagosomes. N, nucleus; M, mitochondria. **C**: OC3/P cells were treated with SAHA, or PTX, or a combination of both as described previously (methods as above). The fractions of cells in G1-phase, S-phase and G2/M phase were determined by flow cytometry.

Figure 5B indicates that treatment with SAHA induced the formation of autophagosomes in a concentration-dependent manner, while treatment with 2 μM PTX for 24 h did not induce OC3/P cell autophagy. Combination treatment with SAHA and PTX further enhanced the number of autophagosomes compared with SAHA treatment alone (Figure 5A).

In this study, cell-cycle progression in OC3/P cells treated with SAHA (16 μM) or/and PTX (2 μM) was examined by flow cytometry. Both SAHA and PTX induced OC3/P arrest in the G2/M phase. SAHA combined with PTX increased the cell accumulation in the G2/M phase to (48.7±4.49)%, SAHA or PTX increased the G2/M phase to (40.9 ± 12.66)% and (34.5 ± 10.32)%, respectively, compared with that in the control group (11.9 ± 2.63)%. SAHA combined with PTX increased the cell accumulation in the S phase to (22.02 ± 6.28)%, SAHA or PTX increased the S phase to (25.29 ± 1.17)% and (49.67 ± 5.25)%, respectively, compared with that in the control group (15.42 ± 3.18)%.

### Effects of SAHA combined with PTX on apoptosis

In this experiment, cell autophagy and changes in apoptosis were observed by electron microscopy. As shown in Figure 5, the integrity of the cell membrane was intact and the cell structure exhibited abundant microvilli, round nuclei, evenly distributed chromatin and cytoplasm stretched in a star-shape into the interconnecting space or fused with neighboring cells in the control group. Cells treated with PTX (2 μM) for 24 h did not show any changes typical of apoptosis, but increased cell abnormalities were observed. The number of microvilli on the cell surface decreased or disappeared and was accompanied by cell shrinkage, cytoplasm and chromatin condensation, marginalization (crescent-shaped or capped nuclear envelope) following treatment with SAHA (16 μM) alone or in combination with PTX. The typical changes of apoptosis were more significant in the combination group than in the SAHA alone group.

**Figure 6:**
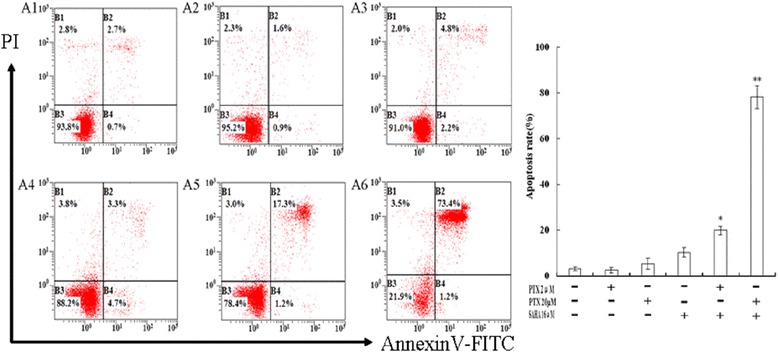
**Annexin V-FITC/PI staining and flow cytometric analysis of apoptosis in OC3/P cells treated with SAHA or/and PTX. A**: A1: Control, A2: 2 μM PTX, A3: 20 μM PTX, A4: 16 μM SAHA, A5: 16 μM SAHA + 2 μM PTX, A6: 16 μM SAHA + 20 μM PTX(methods as above); **B**: Percentages of apoptotic cells are displayed as the mean ± SD of three independent experiments performed in triplicate. *P <0.05 and **P <0.01, indicate significant differences compared with SAHA and PTX single-agent treatments.

Furthermore, the apoptotic changes in each group detected by the Annexin V-FITC/PI assay were consistent with the changes in apoptosis observed by electron microscopy. As shown in Figure 6, no significant apoptosis was observed in OC3/P cells treated with PTX (2 μM) for 24 h. However, significantly higher rates of apoptosis were induced in OC3/P cells by SAHA combined with PTX than by SAHA or PTX alone (*P* <0.01).

### Effects of SAHA combined with PTX on the genes and proteins in OC3/P

**Figure 7:**
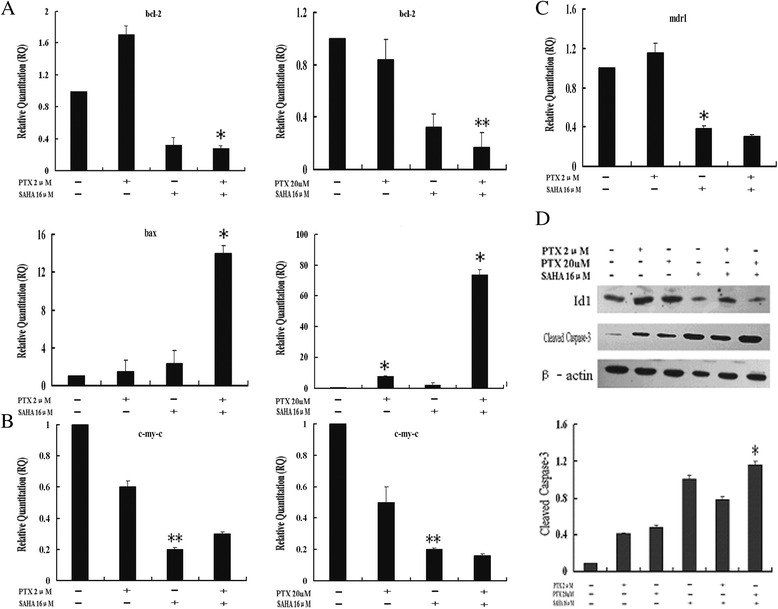
**Effects of SAHA or/and PTX on the expression of intracellular genes and proteins in OC3/P cells. A–C**: Quantitative RT-PCR analysis of *bcl-2*, *bax*, *c-myc* and *mdr1* gene expression in OC3/P cells treated with SAHA or/and PTX. *P <0.05 and **P <0.01 indicate significant differences compared with the control group and drug along group. **D**: Western blot analysis of caspase-3 and Id1 protein expression in OC3/P cells treated with SAHA or/and PTX. *P <0.05 indicate significant differences compared with the control group and drug along group.

As indicated in Figure 7A, a significant decrease in *bcl-2* expression and a parallel increase in bax expression was observed following SAHA + PTX treatment compared with control group, SAHA or PTX alone (*P* <0.05). As shown in Figure 7B, a downregulation in *c-myc* expression in OC3/P cells was observed following treatment with 16 μM SAHA for 24 h. This effect was exacerbated by treatment with 16 μM SAHA combined with 20 μM PTX (*P* <0.05). Moreover, an increase in *mdr1* expression was observed in cells treated with 2 μM PTX for 24 h, with a parallel decrease in *mdr1* expression observed following treatment with SAHA alone or in combination with PTX (Figure 7C).

In addition, Western blot analysis revealed that combined treatment with SAHA and PTX increased the expression of cleaved caspase-3 compared with that observed following SAHA or PTX treatment alone. Treatment with SAHA or PTX alone or in combination reduced the expression of ID1 protein (Figure 7D).

## Discussion

In this study, we first compared differences in OC3 and its paclitaxel-resistant cell line, OC3/P, in terms of morphology and doubling-time. The results showed that paclitaxel-resistant ovarian cancer cells have high atypia, a long doubling-time compared with the characteristics of the paclitaxel-sensitive cells. These observations are consistent with the known difficulties in the treatment of paclitaxel-resistant ovarian cancer and the poor prognosis in such patients. The occurrence of drug resistance in malignant tumors tends to cross-resistance to a variety of chemotherapic drugs, thereby reducing their curative effects. Previous reports have shown that OC3/P exhibits obvious cross-resistance to Topotecan [24]. The RI of OC3/P cells used in this experiment was 10.15, with *mdr1* gene expression nearly 100 times greater than that in OC3 cells (Figure 1). Further studies showed that SAHA exerted an obviously adverse effect on the growth of OC3 cells and the drug-resistant OC3/P cells; however, there was no statistical difference in this effect between the two cell lines (Figure 2). These results suggested that OC3/P is not cross-resistant to SAHA, indicating the potential for its use in OC3/P treatment.

OC3/P cells were pretreated with different concentrations of SAHA for 24 h followed by treatment with 2 μM PTX for 24 h. Compared with PTX or SAHA alone, PTX combined with SAHA significantly enhanced the inhibition of cell growth and decreased the cell migration capability (Figures 3 and 4), indicating that SAHA increased the sensitivity of OC3/P to PTX. Our data indicated that pretreatment with SAHA for 24 h resulted in cell-cycle arrest in the G2/M phase (Figure 5C) and enhanced the antitumor effects of PTX, thereby representing a potential mechanism underlying these effects. Studies have confirmed that, compared with the treatment of unsynchronized ovarian cancer cells, PTX treatment induces a higher rate of apoptosis in cells synchronized to the G2/M phase [25]. It can also be speculated that pretreatment of cells with SAHA for 24 h effectively induced cell apoptosis and increased cell sensitivity to subsequent drug treatment. In addition, SAHA treatment alone causes a concentration-dependent increase in autophagosomes in OC3/P cells (Figure 5A, B), indicating that SAHA induces autophagy in OC3/P cells. However, Chen. [18] reported that SAHA alone did not induce autophagy in ovarian cancer cells. These inconsistencies may be due to the use of different SAHA concentrations and cell lines. In addition, our study showed that PTX alone did not induce cell autophagy, but when combined with SAHA, cell autophagy was significantly enhanced compared with SAHA alone. These observations indicate that autophagy is involved in the mechanism by which SAHA enhances the effects of PTX. In view of this, the expression of genes and proteins related to cell apoptosis was analyzed. The pro-apoptosis gene *bax* and the anti-apoptosis gene *bcl-2* play important roles in Bcl-2 family protein-regulated cell apoptosis. Our study showed that the SAHA in combination with PTX significantly reduced the expression of *bcl-2* genes and simultaneously enhanced the expression of Bax (Figure 7A); this observation is in accordance with those reported by Charles [22].

The caspase family plays a very important role in apoptosis, with the cysteine aspartic acid protease caspase-3 as a key molecule that transmits apoptotic signals in multiple signaling pathways [26]. Our research showed that the caspase-3 protein expression in cells increased after treatment with PTX in combination with SAHA. C-myc can stimulate unlimited cell proliferation, promote cell division, and is also involved in apoptosis. This molecule is closely associated with the occurrence and development of a wide variety of tumors [27,28]. Our study showed that treatment of OC3/P cells with SAHA alone for 24 h significantly reduced *c-my-c* gene expression (Figure 7B); however, in combination with low dose PTX (2 μM), no statistical difference was observed in the changes in *c-myc* observed in response to SAHA treatment alone. In contrast, intracellular *c-myc* expression decreased compared with SAHA alone (*P* <0.05) when combined with high PTX concentrations (up to 20 μM). These observations indicate that mechanism underlying apoptosis induced by SAHA alone or combined with PTX is also associated with changes in *c-myc* expression.

It has been speculated that paclitaxel resistance is mainly related to overexpression of the multi-resistance gene, *mdr1*, tubulin mutations and *bcl-2* expression disorders [29-33]. Our study showed a significant decrease in intracellular *mdr1* gene expression in OC3/P cells treated with SAHA alone for 24 h compared with that in the control group (Figure 7C). Furthermore, in this study, SAHA reduced intracellular *bcl-2* gene expression, which not only represents a mechanism of apoptosis-induction, but may also be involved in the reduction of drug resistance. This indicates that SAHA has the potential to reverse paclitaxel resistance in ovarian cancer. In addition, *mdr1* expression was upregulated in OC3/P treated with PTX alone (2 μM for 24 h) (Figure 7C), indicating that low doses of PTX, not only had no apoptotic effects, but also enhanced the resistance of these cells. These results may also account for the lack of significant effects on cell survival, migration and apoptosis observed in OC3/P treated with PTX alone (2 μM for 24 h). Treatment with PTX (2 μM) alone was associated significantly increased intracellular nuclear abnormalities, which were indicative of enhanced cell resistance. Taken together, these observations indicate that in the combined SAHA and PTX treatment of paclitaxel-resistant ovarian cancer cells, SAHA pretreatment provides an enhanced antitumor effect.

Inhibitors of differentiation or inhibitors of DNA-binding (Id) proteins are widely expressed transcriptional factor with four subtypes: Id1, Id2, Id3 and Id4. These proteins exert negative regulatory effects on cell growth and tissue-specific differentiation by inhibiting further differentiation and maturation after cell division, affecting cell-cycle regulation and promoting proliferation of vascular endothelial cells to promote invasion and metastasis by malignant tumor. Studies have shown that Id1 is highly expressed in ovarian cancer, thus promoting proliferation and inhibiting apoptosis and differentiation of ovarian cancer cells [34,35]. Moreover, Maw found that the expression of Id1 was related positively to the clinical stage of ovarian cancer patients, with higher expression associated with more obvious tumor angiogenesis and poorer prognosis [36]. It has been reported [37] that trichostatin (TSA) effectively inhibits in vitro proliferation and induces anti-differentiation of the sensitive ovarian cancer cell line A2780 via a mechanism that involves downregulation of Id1 protein expression. TSA and SAHA are both HDACIs; therefore, it can be speculated that SAHA has the potential to induce anti-differentiation of paclitaxel-resistant ovarian cancer cells. This study showed that SAHA alone or in combination with PTX effectively reduced the intracellular expression of Id1 protein. Light and electron microscopy investigations also revealed that SAHA (4 μM and 16 μM) treatment of OC3/P wasassociated with changes in nuclear shape, mostly round, cytoplasm increases, microvilli on the cell membrane does not reduce or increase, hence indicating that the induction of anti-differentiation represents another mechanism by which SAHA exerts antitumor activity and enhances the sensitivity of OC3/P cells to PTX. However, treatment with a high concentration of SAHA (64 μM) stimulated apoptosis and autophagy predominantly, therefore, the optimal SAHA dose regimen required to induce anti-differentiation in resistant ovarian cancer cell lines requires further exploration.

In conclusion, SAHA inhibits growth of paclitaxel-resistant ovarian cancer cells, diminishes migration capacity, causes cell-cycle arrest, induces apoptosis and autophagy. In addition, SAHA has the potential to reverse ovarian cancer drug resistance and induce inverse differentiation. These effects are enhanced by pretreatment with SAHA in a combined SAHA and PTX regimen.

## Methods

### Reagents and drugs

The HDACI suberoylanilide hydroxamic acid (SAHA, vorinostat) was provided by Selleck Chemicals (Houston, TX, USA). Paclitaxel was obtained from Bristol-Myers Squibb (Princeton, NJ, USA). RPMI 1640 medium was purchased from Gibco BRL (Grand Island, NY, USA). Thymidine and DMSO were purchased from Sigma-Aldrich (St. Louis, MO, USA). A cell apoptosis kit was purchased from Beijing Baosai Technological Corporation (Beijing, China).CCK-8 was purchased from KEYGEN BIOTECH (Nanjing, China). Anti-cleaved caspase-3 was obtained from Cell Signaling Technology (Beverly, MA, USA). Anti-ID1 was purchased from Santa Cruz Biotechnology (Santa Cruz, CA, USA).

SAHA was dissolved in dimethyl sulfoxide (DMSO) and stored at −70°C until used. SAHA and PTX were diluted to appropriate concentrations in culture medium 1640 and the final concentration of DMSO was less than 0.1% (vol/vol).

### Cell lines and cell culture

OC3 and the derived paclitaxel-resistant cell line, OC3/P, were obtained from the Department of Obstetrics and Gynecology of Beijing Shijitan Hospital of Capital Medical University (China). The paclitaxel-resistant cell line, OC3/P, was induced from OC3 cells by repeat exposure to 300 μg/mL paclitaxell for approximately 2 h each time over a period of 10 months. The cells were cultivated as monolayers in RPMI 1640 medium with 10% heat-inactivated fetal calf serum (HI FCS; PAA Laboratories Gmb) at 37°C in a 5% CO_2_ atmosphere. The culture medium was replaced every 2 days.

### Cell morphology observations with light and electron microscopy

For light microscopy, exponentially growing cells (OC3 and OC3/P) were transferred to 6-well plates and cultured at 37°C in a 5% CO_2_ atmosphere. When the cells were 60% to 70% confluent, the cells were rinsed twice with PBS and the supernatant was discarded. Then, cells were fixed in 4% paraformaldehyde for 20 min and stained by the Wright-Giemsa method.

Transmission electron microscopy (TEM) was used to confirm the morphological features of the induction of apoptosis and autophagy of OC3/P treated with SAHA or PTX alone or in combination by examination of the alterations in the subcellular structures. Cultured cells were fixed and prepared for TEM as described previously [38]. Representative areas were chosen for ultrathin sectioning and photographs were taken with the H-7650 transmission electron microscope (Hitachi Limited, Tokyo, Japan).

### Doubling-time assays and resistance index (RI)

OC3 and OC3/P cells in the exponential phase were digested separately using 0.25% trypsin and harvested. Single-cell suspensions were prepared. OC3 and OC3/P cells were counted separately using a hemocytometer, added to 96-well microtiter plates (5 × 10^3^ cells/well) and cultured at 37°C under 5% CO_2_. Each cell line was tested in triplicate CCK-8 assays every 24 h for 6 days. Two hours before measuring the absorbance, 10 μl of the CCK-8 solution was added into each well. Absorbance at 450 nm was measured using the ELX800 microplate reader (BIO-TEK, USA). Three control wells without cells were prepared and the average absorbance of the control wells was subtracted from that of the corresponding sample wells; doubling-time was calculated according to a previously described method [25]. Each experiment was performed in triplicate.

OC3 and OC3/P cells were added separately to 96-well plates (1.2 × 10^4^ cells/well) and cultured at 37°C under 5% CO_2_ for approximately 24 h prior to the addition of drugs. PTX (0.02, 0.2, 2, 20 and 200 μM) was added to OC3 and OC3/P cells and viability was evaluated after 24 h and 48 h using CCK-8 assays as described previously. Resistance index (RI) was equal to the ratio of the inhibitory concentration 50% (IC_50_) values of resistant to sensitive cells. Each experiment was performed in triplicate.

### Cell viability

OC3 and OC3/P cells were incubated in 96-well plates (1.2 × 10^4^ cells/well) for 24 h prior to treatment with various concentrations of SAHA for 48 h and various concentrations of PTX for 24 h. Cell viability was determined using CCK-8 assay.

OC3/P cells (1.2 × 10^4^ cells/well) were plated in triplicate in 96-well plates for 24 h and treated with various drug groups (Control group: serum-free 1640 for 48 h; SAHA group: SAHA 1640 dilution for 24 h followed by serum-free 1640 for 24 h; PTX group: serum-free 1640 for 24 h followed by PTX 1640 dilution for 24 h; SAHA + PTX group: SAHA 1640 dilution for 24 h followed by PTX 1640 dilution for 24 h). The cell viability of each group was determined by CCK-8 as described previously.

### Wound healing assay

Wound healing assays were conducted as a measure of cell migration and invasion capacity. OC3/P cells in the exponential growth phase were trypsinized and plated into 6-well plates (4 × 104 cells/well). After incubation at 37°C under 5% CO_2_ for 24 h, cells were treated with PTX or SAHA alone or in combination (the co treatment is same with the way in cell viability of methods). A wound was made by scraping the cell monolayers with a 200 μl micropipette tip. Photographs were taken immediately and after 12 h, 24 h and 36 h using an IX70 microscope (OLYMPUS, Japan).

### Flow cytometric analysis of cell-cycle apoptosis

At approximately 40% confluence, 8 mM thymidine was added to OC3/P cells cultured in 6-well plates. After 15 h, the cells were rinsed twice with PBS and the culture medium was replaced with fresh medium (RPMI 1640 medium with 10% HI FCS). After a further 11 h, the cells were treated with thymidine for an additional 15 h. Then, the cells were treated with SAHA or PTX alone or in combination and harvested at the indicated time. The cells were washed with PBS, fixed in 70% ethanol, and stored at 4°C overnight. The fixed cells were washed twice in PBS and the supernatant was discarded. After resuspension in 100 μl PBS containing RNase A (1 mg/ml, Sigma), cells were incubated for 30 min at 37°C. The samples were stained with 400 μl propidium iodide (PI) and then detected using a Cytomics FC500 flow cytometer (Beckman Coulter, Brea, CA, USA). Cell-cycle distribution was calculated using CXP Software (Beckman Coulter), with the number of gated cells in G1, S and G2 phase presented as a percentage. Each experiment was performed in triplicate.

After incubation with SAHA or PTX alone or in combination, OC3/P cells were harvested at the indicated times. The cells (at least 1.0 × 10^6^ events) were rinsed twice with PBS and the supernatant was discarded. The cells were then resuspended in 200 μl of buffer. Annexin V-FITC (10 μl) was added to the cell suspension, mixed slightly and maintained at room temperature for 15 min or at 4°C for 30 min (in darkness). Subsequently, buffer (300 μl) and PI (5 μl) were added to determine that the apoptotic rate using a Cytomics FC500 flow cytometer (Beckman Coulter) over a 1 h period. The percentage of apoptotic cells in each quadrant was calculated using CXP Software. Each experiment was performed in triplicate.

### Quantitative real-time polymerase chain reaction (quantitative RT-PCR)

Total RNA isolation and cDNA synthesis from OC3/P cells treated with SAHA or PTX alone or in combination were performed using Trizol and the Takara Reverse Transcriptase kit (Takara, Otsu, Japan), respectively, according to the manufacturer’s instructions. The cDNA encoding the indicated genes was amplified with the following specific primers:

bcl-2, 5’-ATGTGTGTGGAGAGCGTCAAC-3’ and 5’-AGAGACAGCCAGGAGAAATCAAAC-3’; bax, 5’-GACGGCAACTTCAACTGGG-3’ and 5-CCTGGATGAAACCCTGAAGC-3’c-my-c, 5’-CTGCGACGAGGAGGAGAA-3’ and 5’-CCGAAGGAGAAGGGTGT-3’; mdr1, 5’-ATAATGCGACAGGAGATAGGC-3’ and 5’-ATGTTGCCATTGACTGAAAGAA-3’, actin, 5-TGGCACCCAGCACAATGAA-3 and 5-CTAAGTCATAGTCCGCCTAGAAGCA-3. Real-time PCR was performed using the UltraSYBR Mix (CWBIO, Beijing, China) with the Mx3000p Real-Time PCR System (Stratagene, La Jolla, CA, USA) using the following thermal cycling conditions: 10 min at 95°C followed by 40 cycles of 10 s at 95°C, 30 s at 60°C, and 30 s at 72°C. Data were analyzed according to the 2 ^–ΔΔCt^ method.

### Western blot analysis

After OC3/P cells were treated with SAHA or PTX alone or in combination, they were harvested and lysed. Then total protein concentrations of cell lysates were determined by the BCA Protein Assay kit (Thermo Fisher Scientific, Waltham, MA, USA). Protein samples (60 μg) were separated by 12% SDS-PAGE and transferred onto a PVDF membrane. The membranes were incubated for 1 h in TBST buffer containing 5% skimmed dried milk to block non-specific binding before incubation with the diluted primary antibody overnight at 4°C with gentle shaking. Subsequently, membranes were incubated with the secondary antibody for 1 h at room temperature. The membrane was washed three times in TBST, for 5 min each time, then treated with mixture of chemiluminescence substrate liquid A and liquid B (1:1 ratio) for 5 min in darkness.

### Statistical analysis

The experimental data are shown as the mean ± SD. SPSS18.0 software (Chicago, USA) was used to perform statistical analysis. Statistical comparisons were made using ANOVA, or t-test. A *P* value <0.05 was considered to indicate statistical significance.

## Abbreviations

OC3: Paclitaxel-sensitive ovarian cancer cell line
OC3/P: Paclitaxel-resistant ovarian cancer cell line
SAHA: Suberoylanilide hydroxamic acid
PTX: Paclitaxel
RI: Resistance index
IC50: Half-inhibition concentration
PI: Propidium iodide
